# Supplementary material for: The Pain Divide: a cross-sectional analysis of chronic pain prevalence, pain intensity and opioid utilisation in England
Source: BMJ Open. 2018 Sep 11;8(7):e023391. doi: 10.1136/bmjopen-2018-023391 (PMC6144392; doi:10.1136/bmjopen-2018-023391)
Supplement: Author's manuscript [file bmjopen-2018-023391.draft_revisions.pdf]

BMJ Open

BMJ Open is committed to open peer review. As part of this commitment we make the peer review history of every article we publish publicly available.

When an article is published we post the peer reviewers' comments and the authors' responses online. We also post the versions of the paper that were used during peer review. These are the versions that the peer review comments apply to.

The versions of the paper that follow are the versions that were submitted during the peer review process. They are not the versions of record or the final published versions. They should not be cited or distributed as the published version of this manuscript.

BMJ Open is an open access journal and the full, final, typeset and author-corrected version of record of the manuscript is available on our site with no access controls, subscription charges or pay-per-view fees (http://bmjopen.bmj.com).

If you have any questions on BMJ Open's open peer review process please email info.bmjopen@bmj.com

BMJ Open

The Pain Divide: A cross-sectional analysis of chronic pain prevalence, pain intensity, and opioid utilisation in England

Journal:	*BMJ Open*
Manuscript ID	bmjopen-2018-023391
Article Type:	Research
Date Submitted by the Author:	07-Apr-2018

Complete List of Authors:	Todd, Adam; Newcastle University<br>Akhter, Nasima; Durham University, Wolfson Research Institute for Health and Wellbeing<br>Cairns, Joanne; Newcastle University, Institute of Health and Society<br>Kasim, Adetayo; Durham University, Wolfson Research Institute for Health and Wellbeing<br>Walton, Nick; Newcastle University, Institute of Health and Society<br>Ellison, Amanda; Durham University<br>Chazot, Paul; Durham University<br>Eldabe, Sam; South Tees NHS Trust<br>Bambra, Clare; Newcastle University

Keywords:	PAIN MANAGEMENT, PUBLIC HEALTH, Health policy < HEALTH SERVICES ADMINISTRATION & MANAGEMENT

For peer review only

SCHOLARONE™
Manuscripts

The Pain Divide: A cross-sectional analysis of chronic pain prevalence, pain intensity, and opioid utilisation in England

Adam Todd*[1,2,3], reader in pharmaceutical public health adam.todd@newcastle.ac.uk

Nasima Akhter[2,4], assistant professor (research) nasima.akhter@durham.ac.uk

Joanne-Marie Cairns[1,2,5], lecturer in public health, j_m_cairns@hotmail.co.uk

Adetayo Kasim,[2,4], associate professor (research) a.s.kasim@durham.ac.uk

Nick Walton[1,2,3], research associate, n.walton2@newcastle.ac.uk

Amanda Ellison[4,6], reader in psychology amanda.ellison@durham.ac.uk

Paul Chazot[4,7], associate professor in pharmacology paul.chazot@durham.ac.uk

Sam Eldabe[1,8], consultant anesthesia and pain medicine Sam.Eldabe@stees.nhs.uk

Clare Bambra[1,2], professor of public health, clare.bambra@newcastle.ac.uk

[1]Institute of Health and Society, Faculty of Medical Sciences, Newcastle University, Baddiley-Clark building, Newcastle upon Tyne, NE2 4AX, UK.

[2]Fuse – the UKCRC Centre for Translational Research in Public Health, UK.

[3]School of Pharmacy, Faculty of Medical Sciences, Newcastle University, King's Road, Newcastle upon Tyne, NE1 7RU, UK.

[4]Wolfson Research Institute for Health and Wellbeing, Durham University, Queen's Campus, Stockton-on-Tees, TS17 6BH, UK.

[5] School of Public Health Midwifery and Social Work, Canterbury Christchurch University, Canterbury, CT1 1QU, UK

[6]Department of Psychology, Durham University, South Road, Durham, DH1 3LE, UK.

[7]Department of Biosciences, Durham University, South Road, Durham, DH1 3LE, UK.

[8]Department of Pain and Anaesthesia, The James Cook University Hospital, Marton Rd, Middlesbrough, TS4 3BW, UK.

*Dr. Adam Todd is the corresponding author; adam.todd@newcastle.ac.uk +44 (0) 191 208 2355

For peer review only - http://bmjopen.bmj.com/site/about/guidelines.xhtml

Abstract

Objectives

Our central research question was, in England, are geographical inequalities in opioid use driven by health need (pain)? To answer this question, our study examined: (1) if there are regional inequalities in rates of chronic pain prevalence, pain intensity and opioid utilisation in England; (2) if opioid use and chronic pain are associated after adjusting for individual and area level confounders.

Design

Cross-sectional study design

Setting

England

Primary and secondary outcome measures

Chronic pain prevalence, pain intensity, and opioid utilization

Participants

Participant data relating to chronic pain prevalence, pain intensity, and opioid usage data were obtained at local authority level from the Health Survey for England (n= 5711 respondents who completed this survey).

Methods

Regional and local authority data were mapped, and a generalised linear model was then used to explore the relationships between the data. The model was adjusted to account for area and individual level variables.

For peer review only

Results

There were geographical variations in chronic pain prevalence, pain intensity, and opioid utilisation across the English regions – with evidence of a 'pain divide' between the North and the South, whereby people in the North of England more likely to have 'severely limiting' or 'moderately limiting' chronic pain. The intensity of chronic pain was significantly, and positively associated with the use of opioid analgesics.

Conclusions

There are geographical differences in chronic pain prevalence, pain intensity, and opioid utilisation across England – with evidence of a 'pain-divide'. Given the public health concerns associated with the long-term use of opioid analgesics – and their questionable activity in the management of chronic pain – more guidance is need to support prescribers in the management of long chronic pain so the initiation of opioids can be avoided.

Keywords: opioids, chronic pain, public health, appropriate prescribing

Word count of main manuscript: 3327

For peer review only

For peer review only - http://bmjopen.bmj.com/site/about/guidelines.xhtml

Strengths and limitations of this study

- This study is unique in that we explored the association of opioid utilisation and chronic pain
- We adjusted for individual (e.g. age and sex) and area-level confounders (e.g social deprivation) in our model.
- We did not distinguish between weak and strong opioid in our analysis, nor did we consider dose of opioid

For peer review only

For peer review only - http://bmjopen.bmj.com/site/about/guidelines.xhtml

Introduction

Chronic pain is a worldwide problem, and the burden it places on our society is increasing: in the US, the annual cost of chronic pain – through direct and indirect effects – is estimated to exceed $500 billion, while in the UK estimates suggest it costs around £12 billion per year to the economy.[1, 2] To manage the symptoms associated with chronic pain, some treatment strategies rely on the use of opioid analgesics, although there are very few studies to support their long-term effectiveness.[3-5] In addition, prolonged use of opioids can also have adverse consequences; this can include sleep disturbances, endocrine disorders, reduced immune function and increased pain through opioid-induced hyperalgesia.[6-10]

Despite these well-acknowledged shortcomings, the prescribing of opioid analgesics continues to increase at a significant rate.[11-12] Indeed, figures from the UK show that, in 2014, there were around 23 million prescriptions written for opioid analgesics, at a cost of around £322 million.[13] Given this increased use, (and the well-established problems associated with efficacy, tolerance and adverse effects) the inappropriate prescribing – and misuse – of opioid analgesics is becoming a significant public health concern.[14] This problem is also mirrored in other countries, such as the US, where the death rate from opioid misuse has, in the last 15 years, quadrupled – giving rise to the so-called 'opioid epidemic'.[15]

In England, there is significant geographical variation in opioid prescribing – with more people in the North of England prescribed opioids – at a greater cost – compared to the rest of England. For example, the North of England (population of 15 million) accounts for approximately 33 per cent of the total costs of analgesics, compared to London (population of 8.2 million), that accounts for only around 8 per cent.[12] It is not clear, however, if this variation is related to 'inappropriate prescribing' or the varying health need of the population

For peer review only

For peer review only - http://bmjopen.bmj.com/site/about/guidelines.xhtml

(*i.e.* more people in the North of England have pain, hence the prescribing of opioids is higher). It is well documented, though, that mortality and morbidity rates are higher in the North of England, particularly in the North-East region compared to the rest of England: an observation known as the North South health divide.[16]

Northern England (commonly defined as the North East, North West and Yorkshire and Humber regions) has persistently had higher all-cause mortality rates than the South of England, with people in the North consistently found to be less healthy than those in the South - across all social classes and amongst men and women.[17] Since 1965, this has amounted to 1.5 million excess premature deaths.[18] Further, the gap in average life expectancy gap between the North and the South of England is 2 years.[16] Although England is not alone in experiencing such spatial health inequalities, the divide in England is one of the largest in Europe – greater, for example, than those between the former East and West of Germany.[19] Social science suggests that the reasons for the contemporary health divide are both compositional and contextual.[16] Compositional factors include demographic factors (e.g. age, sex, marital status) and socio-economic status (e.g. employment, income, education, occupation), as well as health behaviours (e.g. smoking, alcohol, physical activity). In the case of pain, other compositional factors will include co-morbidities such as depression or anxiety. Contextual factors include the physical (e.g. air pollution or contaminated land),[20] social (e.g. place based stigma or social networks or access to services such as GPs)[21] and economic (e.g. area-level deprivation, local job availability) environments.[22]

Given the North South health divide and public health concerns associated with the inappropriate and long-term use of opioid analgesics, it is vitally important then to explore whether the prescribing of opioid analgesics across England reflects inequalities in the health needs of the population (pain) or if there is an issue of 'inappropriate' medication prescribing

For peer review only - http://bmjopen.bmj.com/site/about/guidelines.xhtml

or utilisation. Our central research question therefore was, in England, are geographical inequalities in opioid use driven by health need (pain)? To answer this question, our study examined: (1) if there are regional inequalities in rates of chronic pain prevalence, pain intensity and opioid utilisation in England; (2) if opioid use and chronic pain are associated after adjusting for individual and area level confounders.

Methods

Data and Variables

Local authority level Health Survey for England (HSE) data were obtained from the National Centre for Social Research, which contains anonymised individual-level data and a geographic identifier (Local Authority District which are large administrative areas used by local government in England and have the responsibility for health and social care, education, transport and so forth). The HSE is an annual survey designed to be representative using a stratified random sample. Each year there is a focus on a particular population group, condition or disease. In 2011, one particular focus of the HSE was detailing chronic pain: as part of the wider survey, participants were asked:

- Whether they were currently troubled by pain or discomfort?
- Whether they had this pain or discomfort for more than 3 months?

If the respondent answered yes to both questions, they were categorised as experiencing chronic pain. Once it was established that participants had chronic pain, they were then asked a further three questions:

- How would you rate your pain right now, on a scale from 0 to 10, where 0 is no pain and 10 is pain as bad as it could be?

For peer review only - http://bmjopen.bmj.com/site/about/guidelines.xhtml

- In the last three months, how would you rate your worst pain, on a scale from 0 to 10, where 0 is no pain and 10 is pain as bad as it could be?
- On average, in the last three months, how would you rate your pain on a scale from 0 to 10, where 0 is no pain and 10 is pain as bad as it could be?

The answers to these questions were then used to compute a variable on pain intensity on a scale of 0 to 4, indicating 'grade 0 - no intensity (i.e. no chronic pain)', 'grade 1 – low intensity', 'grade 2 – high intensity', grade 3 – moderately limiting', 'grade 4 – severely limiting'. This grading was based upon the 3-item Graded Chronic Pain (GCP)-Pain Catastrophizing Scale (PCS).[23]

Opioid usage data was also contained in the 2011 HSE; this used the British National Formulary (BNF) classification code for opioid analgesic medications. Socio-demographic variables included were age, sex, marital status, highest educational qualifications, occupational classifications, household income quintile. Health related data included self-assessed general health status (very good; good; fair; bad; very bad), presence of mental health disorder (yes / no), anxiety levels (not anxious or depressed; moderately anxious or depressed; extremely anxious or depressed), and ranking of happiness on a 0 to 10 scale. Area-level deprivation data included the Index of Multiple Deprivation (IMD) 2010 obtained from the HSE. The IMD produces a ranking of areas in England based on relative local scores for: income, employment, health, education, crime, access to services and living environment. IMD was included because there is a strong relationship between area level deprivation and mortality and morbidity – with the most deprived neighbourhoods in England experiencing life expectancy nine and six years less for men and women respectively than those that are the least deprived.[24]

For peer review only - http://bmjopen.bmj.com/site/about/guidelines.xhtml

The English regions were classified as the North (North East, North West, Yorkshire & the Humber) and the South (London, East of England, West Midlands, East Midlands, South East and South West). This study used individual level HSE data and therefore HSE survey weights applicable for individual level data were used.

Data Analysis

Chronic pain prevalence, pain intensity, and opioid usage were mapped using Adobe Illustrator with local and regional boundaries downloaded from the Office for National Statistics. In the HSE, opioid use was described as a binary variable (a yes or no response), and was used as an outcome variable to examine the association between opioid use and factors associated with it. The complete dataset with no missing values (n = 5711) was used in our analysis. Variables that showed significantly bivariate association were included in the initial model. Apart from the presence of chronic pain and pain intensity, the initial model included age, sex, marital status, highest educational qualification, occupational level, household income quintiles, general health status, mental health disorders, anxiety levels, and happiness scale. A generalised linear model with binomial distribution and logit link was used to examine the associations between opioid use and chronic pain, adjusted for individual and area level covariates. Survey weight was applied to the model. The most parsimonious model was obtained by using likelihood ratio test statistics to ensure there was no significant loss of information. To support the spatial analysis of a 'pain-divide' between the North and the South of England, the pain intensity data were analysed using a generalised logit model to simultaneously analyse the four logit models resulting from the five levels of the pain intensity data (no pain, low intensity, high intensity, moderately limiting and severely limiting). Although the pain intensity is ordinal, the proportional odds model is both intuitively and statistically not appropriate because of the assumption of the common odds

For peer review only - http://bmjopen.bmj.com/site/about/guidelines.xhtml

between the levels of pain intensity data. Survey weight was used in all analyses to ensure generalization of findings.

This study was undertaken and reported according to the strengthening the reporting of observational studies in epidemiology (STROBE) recommendations.[25]

Patient and Public Involvement

As this study involved secondary data analysis from the HSE, patients or the public were not involved in the design, or delivery of this research.

Ethical approval

Ethical approval of this work was not required, as the study used non-patient identifiable secondary data; patients were not actively involved in this research.

Results

Regional inequalities in the prevalence of chronic pain, pain intensity and opioid use in England

The prevalence of chronic pain was 39.6% in the North of England, compared to the 37.5% in the South of England, as shown in Table 1, and visually in Figure 1. In terms of the nine English regions, the prevalence of chronic pain was highest in the North East, and lowest in London (43.1% vs. 29.0%). In terms of pain intensity, 10.2% of people living in the South had 'moderately limiting' or 'severely limiting' chronic pain, while, in the North, 13.9% of people had 'moderately limiting' or 'severely limiting' chronic pain. People in the North were also more likely to experience 'moderately limiting' or 'severely limiting' pain than those in the South: the odds of severely limiting pain were 32% higher in the North than in

For peer review only - http://bmjopen.bmj.com/site/about/guidelines.xhtml

For peer review only

the South; similarly, the odds of 'moderately limiting' pain were 37% higher in the North than the South, as shown in Table 2. In addition to differing pain levels in the North and South English regions, there were also observed differences in anxiety and self-reported general health: anxiety levels in the North were 27.3%, compared to 25.7% in the South, while for self-reported general health, 7.6% and 5.5% of people living in the North and South respectively were reported to have 'bad' or 'very bad' health status. Although chronic pain prevalence was similar in the North and South of England (39.6% and 37.5%, respectively), opioid use was somewhat higher in the North (3.0%), compared to the South (1.9%). Furthermore, the use of opioids were higher in the North of England for people with 'severely limiting' chronic pain (18%), compared to people in the South (11%), as illustrated by Figure 2.

For peer review only

For peer review only - http://bmjopen.bmj.com/site/about/guidelines.xhtml

Table 1. Characteristics of the study population

Variable		South % (n)	North % (n)	Overall % (n)
Age group	Median (25th, 75th percentile)	45 (32, 60)	45 (32, 60)	45 (32, 60)
Sex	Male	44.1 (1718)	44.7 (812)	44.3 (2530)
	Female	55.9 (2178)	55.3 (1003)	55.7 (3181)
Opioid use	No	98.1 (3821)	97.0 (1760)	97.7 (5581)
	Yes	1.9 (75)	3.0 (55)	2.3 (5581)
Chronic Pain	No	62.5 (2435)	60.4 (1097)	61.8 (3532)
	Yes	37.5 (1461)	39.6 (718)	38.2 (2179)
Pain intensity	None	62.5 (2435)	60.4 (1097)	61.8 (3532)
	Low intensity	1.77 (65)	1.5 (27)	1.6 (92)
	High intensity	25.6 (998)	24.2 (439)	25.2 (1437)
	Moderately limiting	3.5 (137)	4.7 (85)	25.2 (1437)
	Severely limiting	6.7 (261)	9.2 (167)	7.5 (428)
Anxiety grades	Not anxious	74.3 (2894)	72.7 (1319)	73.8 (4213)
	Moderate	23.5 (917)	24.4 (443)	23.8 (1360)
	Extreme	2.2 (85)	2.9 (53)	2.4 (138)
Income quintiles	Lowest	14.2 (552)	17.7 (322)	15.3 (874)
	Second lowest	18.2 (708)	25.1 (455)	20.4 (1163)
	Middle	19.9 (775)	20.6 (374)	20.1 (1149)
	Second highest	22.7 (884)	20.2 (367)	21.9 (1251)
	Highest	25.1 (977)	16.4 (297)	22.3 (1274)
Occupation	Managerial and professional	40.2 (1566)	34.2 (620)	38.3 (2186)
	Intermediate	25.2 (982)	21.5 (390)	24.0 (1372)
	Routine and manual	31.0 (1209)	40.9 (743)	34.2 (1952)
	Other	3.6 (139)	3.4 (62)	3.5 (201)
Educational qualifications	No qualifications	17.3 (674)	21.8 (395)	8.7 (1069)
	Foreign/ other	1.4 (54)	1.5 (27)	1.4 (81)
	NVQ1 or equivalent	4.0 (155)	4.7 (86)	4.2 (241)
	NVQ2 or equivalent	22.2 (864)	22.0 (399)	22.1 (1263)
	NVQ3/ A level equivalent	15.6 (608)	15.4 (279)	15.5 (887)
	Higher education	11.9 (462)	12.2 (222)	12.0 (684)
	NVQ4/ Degree or equivalent	27.7 (1079)	22.4 (407)	26.0 (1488)
General Health	Very good	34.7 (1353)	31.5 (571)	33.7 (1924)
	Good	44.1 (1719)	42.8 (776)	43.7 (2495)
	Fair	15.6 (607)	18.2 (330)	16.4 (937)
	Bad	4.2 (165)	5.3 (96)	4.6 (261)
	Very bad	1.3 (52)	2.3 (42)	1.6 (94)
Happiness scale	Median (25th, 75th percentile)	8 (7,9)	8 (7,9)	8 (7, 9)

For peer review only

For peer review only - http://bmjopen.bmj.com/site/about/guidelines.xhtml

Table 2. Estimated odds ratios from generalised logit analysis of different pain intensities between North and South of England adjusting for age, gender and level of qualifications.

Variables	Categories	'Severely limiting' Vs 'No Pain'	'Moderately limiting' Vs 'No Pain'	'High Intensity' Vs 'No Pain'	'Low Intensity' Vs 'No Pain'
Intercept		0.011 (0.007,0.018)	0.010 (0.006,0.018)	0.097 (0.076,0.124)	0.004 (0.002,0.010)
Region	North	1.323 (1.063,1.645)	1.374 (1.035,1.823)	0.977 (0.852,1.120)	0.954 (0.604,1.507)
	South	Ref	Ref	Ref	Ref
Age		1.042 (1.035,1.050)	1.033 (1.024,1.042)	1.030 (1.025,1.034)	1.036 (1.022,1.054)
Gender	Female	1.137 (1.020,1.267)	1.221 (1.059,1.408)	1.183 (1.108,1.262)	0.913 (0.738,1.129)
	Male	Ref	Ref	Ref	Ref
Qualification	None	2.574 (2.069,3.203)	1.408 (1.021,1.943)	1.133 (0.964,1.332)	1.117 (0.660,1.890)
	Foreign/ other qualification	1.188 (0.635,2.222)	0.873 (0.351,2.171)	0.894 (0.574,1.392)	1.373 (0.384,4.911)
	NVQ1 CSE other grade equivalent	1.345 (0.872,2.077)	1.082 (0.587,1.995)	1.199 (0.917,1.569)	0.562 (0.164,1.922)
	NVQ2 GCE O level equivalent	1.057 (0.822,1.358)	1.073 (0.776,1.483)	1.007 (0.868,1.169)	0.748 (0.422,1.326)
	NVQ3 GCE A level equivalent	0.853 (0.621,1.171)	0.744 (0.487,1.138)	0.913 (0.768,1.087)	0.866 (0.459,1.636)
	Higher education below degree	0.718 (0.513,1.005)	0.937 (0.627,1.401)	0.999 (0.836,1.194)	1.162 (0.654,2.065)
	NVQ4/NVQ5/ Degree	Ref	Ref	Ref	Ref

For peer review only

For peer review only - http://bmjopen.bmj.com/site/about/guidelines.xhtml

Association of opioid utilisation and chronic pain after adjusting for individual and area level confounders

Opioid usage was significantly associated with chronic pain intensity (adjusted for age, household income, occupation level, general health and anxiety): in people with higher pain intensities, there were higher odds of opioid use, as illustrated by Table 3. The use of opioids were also positively associated with household income levels: households belonging to the 3rd to 5th (highest) income quintiles had significantly higher odds of using opioids than those at the lowest quintile. In addition, general health status was significantly positively associated with opioid usage: people who reported 'very bad' or 'bad' health status had 14% higher odds, and 6% higher odds of using opioids respectively, compared to those who reported 'very good' health status. Finally, participants who reported extreme anxiety or depression had significantly higher odds of using opioid analgesics, compared to participants who were not anxious.

For peer review only

Table 3. Generalised Linear Model examining associations between opioid use and chronic pain

Variables	Categories	Odds Ratio (Confidence Intervals)
Intercept		0.970 (0.956, 0.985)
Age		1.000 (1.000, 1.000)
Pain grade	Severely limiting	1.078 (1.060, 1.097)
	Moderately limiting	1.036 (1.016, 1.056)
	High intensity	1.022 (1.013, 1.031)
	Low intensity	0.995 (0.968, 1.023)
	No chronic pain	Ref
Income quintile	Highest quintile	1.017 (1.004, 1.030)
	4th	1.018 (1.006, 1.030)
	3rd	1.018 (1.006, 1.030)
	2nd	1.016 (1.004, 1.028)
	Lowest quintile	Ref
Highest qualification	No qualification	1.012 (1.000, 1.024)
	Foreign/ other	1.005 (0.972, 1.039)
	NVQ1 or equivalent	1.002 (0.984, 1.021)
	NVQ2 or equivalent	1.004 (0.994, 1.015)
	NVQ3/ A level equivalent	1.011 (1.000, 1.022)
	Higher education	1.005 (0.992, 1.017)
	NVQ4/ Degree or equivalent	Ref
General Health	Very bad	1.137 (1.102, .174)
	Bad	1.057 (1.035, 1.080)
	Fair	1.022 (1.010, 1.034)
	Good	1.000 (0.992, 1.008)
	Very good	Ref
Anxiety	Extreme	1.015 (0.991, 1.039)
	Moderate	1.008 (1.000, 1.017)
	Not anxious	Ref

For peer review only - http://bmjopen.bmj.com/site/about/guidelines.xhtml

Discussion

This paper is the first to examine geographical inequalities in chronic pain prevalence, pain intensity, and opioid utilisation in England. It is also the first to examine the association between chronic pain intensity and opioid utilisation. We have identified two key findings that may be of importance to healthcare practitioners and policy makers: (1) there are geographical variations in chronic pain prevalence, pain intensity, and opioid utilisation across the English regions – with evidence of a 'pain-divide' with people in the North of England more likely to have higher intensity of pain; (2) opioid utilisation was significantly, and positively associated with pain intensity. The higher prevalence and intensity of pain in the Northern regions, as well as more people to lower education groups may only partly explain the higher rates of opioid usage found there. However, the number of people who used opioids in the survey was too small to support an interaction model between pain intensity and regions or a separate subgroup analysis for each region. These findings suggest the reason why people in the North East of England are prescribed more opioid analgesics than other parts of England is owing to the higher health need (pain). This is in keeping with wider studies of regional inequalities in health [16] and is a potentially important and significant finding given the recent public health concerns associated with opioid analgesics.

While this is the first study to examine the relationship between chronic pain intensity and opioid usage in England, there have been other studies that have explored the geographical variation in opioid prescribing. For example, a recent study by Mordecai and colleagues showed that, at a clinical commissioning group (CCG) level, over a four-year period, there was an increasing trend of opioid prescribing – with more opioid analgesics prescribed in the North of England, compared to the South.[26] Our work builds on these findings, and shows that the increased trend of opioid prescribing is associated with an increase in health need (pain), rather than an 'inappropriate' prescribing trend of opioid analgesics. In addition to

For peer review only - http://bmjopen.bmj.com/site/about/guidelines.xhtml

this, there have been a number of studies that have explored prescribing variation in other parts of the world, such the US,[27, 28] Canada,[29] and Australia;[30] these studies have also showed there is a large geographical variation in prescribing practices of opioid analgesics, and call for guidance to promote good prescribing practices. Our results are timely, and show that, in England, the prescribing of opioid analgesics is largely driven by health need (pain): thus, to develop future strategies going forward, and avoid a potential 'opioid epidemic', as observed in the US, it is important that consideration is given to other ways of managing chronic pain, without the use of opioid analgesics. While opioids may have a role in the short-term management of pain, their long-term use is questionable.[6-10] Currently, national guidelines recommend strong opioids as an option for pain relief for patients with chronic pain, providing they are reviewed annually, and only continued if they are providing on-going pain relief.[31] While this is helpful in some instances, it is often difficult to ascertain, in a clinical setting, if opioid analgesics continue to provide on-going pain relief; patients using opioids are also often reluctant to reduce or stop their opioid medication.[32,33] Studies also show that opioid discontinuation is associated with reducing pain scores; opioid induced hyperalgesia also reduces upon opioid cessation, which can further reduce levels of pain.[34] Given our findings, more needs to be done – at a national level – to support prescribers to manage people who have chronic pain, without the need to initiate opioid analgesics. Another potential that could be potentially used alongside this approach would be to consider how opioids are monitored and stopped in the community. We note the recent attention given to the term 'deprescribing'– a term used to describe the process of reducing or stopping inappropriate medication, with a view to minimising polypharmacy and improving patient outcomes.[35] It would be prudent to suggest that future prescribing strategies for opioids should also include an element of 'deprescribing' to ensure

For peer review only - http://bmjopen.bmj.com/site/about/guidelines.xhtml

that if opioids are to be initiated, patients do not continue to use or be prescribed opioids for chronic pain indefinitely without benefit.

Our findings relating to geographical inequalities in chronic pain are in keeping with research into a number of other health outcomes, such as obesity, diabetes, cancer and cardiovascular disease, where higher rates are reported in the North – and in particular the North East – compared to the other English regions.[16] Our work suggests that the North South health divide could increase in the future unless prescribing practices change because current guidance for using opioids to manage pain means that the North will have a higher burden of side effects in the future. Further, with an ageing population (particularly in the North) and an associated increase in chronic conditions, then we anticipate a further increase in pain and therefore opioid use. Again, given the regional inequalities in the burden of disease, this could exacerbate further the North South divide. This is timely, as the recent Due North report,[18] an independent inquiry, commissioned by Public Health England, to identify actions that can reduce the gap in health between the North and South of England suggests that an urgent holistic approach is needed to ensure that future investment is effective at reducing inequalities. Our study shows that examination of the need for continued opioid prescribing should be considered in any strategies going forward to tackle the poorer health outcomes commonly reported in the North East of England, compared to the rest of the country.

In terms of study limitations, we acknowledge that there are several: firstly, in our analysis we used chronic pain prevalence and pain intensity as the marker for health need. Opioids are also used in the management of other conditions, such as acute post-operative pain, cancer pain, or in the management of opioid substance dependence; clearly, this will have an influence regarding opioid prescribing practices. Also, the analysis does not discriminate between specific opioids, potency of opioid (*e.g.* strong opioids versus weak opioids) or

For peer review only - http://bmjopen.bmj.com/site/about/guidelines.xhtml

opioid dosages. It is also important to consider that geographical scale is important when exploring variation amongst a given area: it is possible that, even at Local Authority level, the opioid prevalence estimates are concealing further geographical patterning since they still contain relatively large populations. A finer scale analysis may, therefore, highlight particular opioid 'hotspots' where opioid prescribing and utilisation is concentrated. Another study limitation is that the HSE data was from 2011, although we note this is the most recent and meaningful data on chronic pain prevalence and pain intensity. Finally, the usual limitations of using cross-sectional data apply to this study meaning that we cannot claim causation only association, nor can we say that this association applies at other geographical scales, only at a regional level. While we believe our results are robust, and have important policy implications, they should be interpreted cautiously in view of our acknowledged limitations.

Conclusion

There are geographical differences in chronic pain prevalence, pain intensity, and opioid utilisation across England – with evidence of a 'pain-divide' with people in the North of England more likely to have 'severely limiting' or 'moderately limiting' chronic pain. In our model, the intensity of chronic pain was significantly, and positively associated with the use of opioid analgesics. Given the public health concerns associated with the long-term use of opioid analgesics – and their questionable activity in the management of chronic pain – more guidance is need to support prescribers in the management of long chronic pain so the initiation of opioid can be avoided. Future opioid prescribing strategies should also consider incorporating deprescribing approaches to ensure when opioids are initiated, their use is regularly monitored, reviewed and, discontinued in the community.

For peer review only - http://bmjopen.bmj.com/site/about/guidelines.xhtml

Figure/Table legends

Figure 1: Prevalence of chronic pain by local authority and English region

Figure 2: Opioid use among participants from the North and South of England according to chronic pain grades

Table 1: Characteristics of the study population

Table 2: Estimated odds ratios from generalised logit analysis of different pain intensities between North and South of England adjusting for age, gender and level of qualifications.

Table 3. Generalised linear model of associations between opioid use and chronic pain

Contributions

AT, CB and SE designed the study, and supervised all stages of the research. AT led the drafting of the manuscript with input from all authors. AK and NA led the statistical analyses; NW cleaned the data, conducted preliminary analyses and commented on the drafts. CB, AT, and JC led on data interpretation. AE and PC informed the initial study design and commented on the analysis, and interpretation. AT is the corresponding author and acts as guarantor of the article.

Competing interests

The authors declare that they have no competing interests.

Funding

This research received no specific grant from any funding agency in the public, commercial or not-for-profit sectors.

For peer review only

Ethical approval and consent to participate

Ethical approval of this work was not required, as the study used non-patient identifiable secondary data; patients were not actively involved in this research.

Acknowledgements

We would like to acknowledge the National Centre for Social Research for supplying us with the sub-national HSE data required for the analysis. The Wolfson Research Institute for Health & Wellbeing supported us financially with a small grant to purchase the data. Chris Orton, Durham University, in Cartography produced the map in this paper, and we thank him for his assistance. Author CB is a member of Fuse. Funding for Fuse comes from the British Heart Foundation, Cancer Research UK, Economic and Social Research Council, Medical Research Council, the National Institute for Health Research, under the auspices of the UK Clinical Research Collaboration, and is gratefully acknowledged (RF150334). The views expressed in this paper do not necessarily represent those of the funders or UKCRC. The funders had no role in study design, data collection and analysis, decision to publish, or preparation of the manuscript.

Data sharing agreement

Unfortunately, we are unable to share our data, as it does not belong to us. We have an agreement with HSCIC (source of our secondary data) that we will delete the data once we are finished using it.

For peer review only - http://bmjopen.bmj.com/site/about/guidelines.xhtml

References:

1. Relieving Pain in America: A Blueprint for Transforming Prevention, Care, Education, and Research. Institute of Medicine (US) Committee on Advancing Pain Research, Care, and Education. Washington (DC): National Academies Press (US); 2011

2. Maniadakis N, Gray A. The economic burden of back pain in the UK. *Pain.* 2000; 84(1): 95-103.

3. Azevedo L, Costa-Pereira A, Mendonça L, Dias C, Castro-Lopes J. A population-based study on chronic pain and the use of opioids in Portugal. *Pain.* 2013; 154(12): 2844-52.

4. Jensen M, Thomsen A, Højsted J. 10-year follow-up of chronic non-malignant pain patients: opioid use, health related quality of life and health care utilization. *Eur J Pain.* 2006; 10(5): 423-33.

5. Stannard C. Opioids for chronic pain: promise and pitfalls. *Curr Opin Support Palliat Care.* 2011; 5: 150-7.

6. Webster L, Choi Y, Desai H, Webster L, Grant B. Sleep-disordered breathing and chronic opioid therapy. *Pain Med.* 2008;9(4):425-32.

7. Wang D, Teichtahl H. Opioids, sleep architecture and sleep-disordered breathing. Sleep Med Rev. 2007;11(1):35-46.

8. Asaad TA, Ghanem MH, Abdel Samee, AM, El–Habiby, MM. Sleep Profile in Patients With Chronic Opioid Abuse: A Polysomnographic Evaluation in an Egyptian Sample. *Addictive Disorders & Their Treatment.* 2011;10(1):21-8.

For peer review only

For peer review only - http://bmjopen.bmj.com/site/about/guidelines.xhtml

9. Martell B, O'Connor P, Kerns R, Becker W, Morales K, Kosten T, *et al.* Systematic review: opioid treatment for chronic back pain: prevalence, efficacy, and association with addiction. *Ann Intern Med.* 2007; 146(2): 116-27.

10. Berna C, Kulich R, Rathmell J. Tapering Long-term Opioid Therapy in Chronic Noncancer Pain: Evidence and Recommendations for Everyday Practice. *Mayo Clin Proc.* 2015; 90(6): 828-42.

11. Zin C, Chen L-C, Knaggs R. Changes in trends and pattern of strong opioid prescribing in primary care. *Eur J Pain.* 2014; 18(9): 1343–51.

12. Murphy E, Spain V. General Practice Prescribing Trends: 2014 Annual Review. London: Cogora, 2015.

13. Team PaM. Prescriptions dispensed in the community: England, 2004 to 2014: Health and Social Care Information Centre; 2015. Available from: http://www.hscic.gov.uk/catalogue/PUB17644/pres-disp-com-eng-2004-14-rep.pdf (last accessed 04.03.2018)

14. Schmidt TD, Haddox JD, Nielsen AE, Wakeland W, Fitzgerald J. Key Data Gaps Regarding the Public Health Issues Associated with Opioid Analgesics. *J Behav Health Serv Res.* 2015; 42(4): 540-53.

15. Calcaterra S, Glanz J, Binswanger IA. National trends in pharmaceutical opioid related overdose deaths compared to other substance related deaths: 1999-2009. *Drug Alcohol Depend.* 2013; 131(3): 263-70.

16. Bambra, C. Health Divides: Where You Live Can Kill You. Policy Press. 2016. ISBN: 978-1447330356.

For peer review only - http://bmjopen.bmj.com/site/about/guidelines.xhtml

17. Dorling D 2010 *Persistent North-South Divides* in Coe, NM Jones A eds The Economic Geography of the UK Sage Publications London.

18. Whitehead M (chair), Bambra C, Barr B, Bowles J, Caulfield R, Doran T, Harrison D, Lynch A, Pleasant S, and Weldon, J. (2014) *Due North: The Independent Inquiry into Health Equity in the North* CLES: Manchester. (http://www.cles.org.uk/publications/due-north-report-of-the-inquiry-on-health-equity-for-the-north/) (last accessed 04.03.2018)

19. Bambra C, Barr B, Milne E. 2014 North and South: addressing the English health divide. *J Public Health (Oxf)*. 2014;36(2):183-6.

20. Bambra C, Cairns JM, Kasim A, Smith J, Robertson S, Copeland A, Johnson K. (2015) This divided land: An examination of regional inequalities in exposure to brownfield land and the association with morbidity and mortality in England. *Health Place.* 2015;34:257-69.

21. Todd A, Copeland A, Kasim A, Husband A, Bambra C. Access all areas? An area-level analysis of the relationship between community pharmacy and primary care distribution, urbanity and social deprivation in England, *BMJ Open*. 2015;5:e007328.

22. Macintyre S, Ellaway A, Cummins S. Place effects on health: how can we conceptualise, operationalise and measure them? *Soc Sci Med.* 2002;55(1):125-39.

23. Sullivan MJL. The Pain Catastrophizing Scale (PCS); user manual. Available at: http://sullivan-painresearch.mcgill.ca/pdf/pcs/PCSManual_English.pdf (last accessed 04.03.2018)

24. Inequality in healthy life expectancy at birth by national deciles of area deprivation: England. Office for National Statistics. Available at: https://www.ons.gov.uk/peoplepopulationandcommunity/healthandsocialcare/healthandlifeex

For peer review only

pectancies/datasets/inequalityinhealthylifeexpectancyatbirthbynationaldecilesofareadeprivationengland (last accessed 04.03.2018)

25. STROBE statements. Available at: https://strobe-statement.org/index.php?id=available-checklists (last accessed 04.03.2018)

26. Mordecai L, Reynolds C, Donaldson LJ, Williams A. Patterns of regional variation of opioid prescribing in primary care in England: a retrospective observational study. *Br J Gen Pract* 2018; DOI: https://doi.org/10.3399/bjgp18X695057.

27. McDonald DC, Carlson K, Izrael D. Geographic variation in opioid prescribing in the U.S. *J Pain.* 2012; 13(10): 988-96.

28. Kuehn BM. CDC: Major disparities in opioid prescribing among states: some states crack down on excess prescribing. *JAMA.* 2014; 312(7): 684-6.

29. Gomes T, Juurlink D, Moineddin R, Gozdyra P, Dhalla I, Paterson M, Mamdani M. Geographical variation in opioid prescribing and opioid-related mortality in Ontario. *Healthc Q.* 2011;14(1):22-4.

30. Degenhardt L, Gisev N, Cama E, Nielsen S, Larance B, Bruno R. The extent and correlates of community-based pharmaceutical opioid utilisation in Australia. *Pharmacoepidemiol Drug Saf.* 2016; 25(5):521-38.

31. SIGN 136. Management of Chronic Pain. Available at: http://www.sign.ac.uk/pdf/SIGN136.pdf (last accessed 04.03.2018)

32. Kennedy LC, Binswanger IA, Mueller SR, Levy C, Matlock DD, Calcaterra SL, Koester S, Frank JW. "Those Conversations in My Experience Don't Go Well": A Qualitative Study

For peer review only - http://bmjopen.bmj.com/site/about/guidelines.xhtml

of Primary Care Provider Experiences Tapering Long-term Opioid Medications. *Pain Med.* 2017; doi: 10.1093/pm/pnx276.

33. Frank JW, Levy C, Matlock DD, Calcaterra SL, Mueller SR, Koester S, Binswanger IA. Patients' Perspectives on Tapering of Chronic Opioid Therapy: A Qualitative Study. *Pain Med.* 2016;17(10):1838-1847.

34. Sullivan MD, Turner JA, DiLodovico C, D'Appollonio A, Stephens K, Chan YF. Prescription Opioid Taper Support for Outpatients With Chronic Pain: A Randomized Controlled Trial. *J Pain.* 2017;18(3):308-318.

35. Scott IA, Hilmer SN, Reeve E, Potter K, Le Couteur D, Rigby D, Gnjidic D, Del Mar CB, Roughead EE, Page A, Jansen J, Martin JH. Reducing inappropriate polypharmacy: the process of deprescribing. *JAMA Intern Med.* 2015; 175(5): 827-34.

For peer review only

For peer review only - http://bmjopen.bmj.com/site/about/guidelines.xhtml

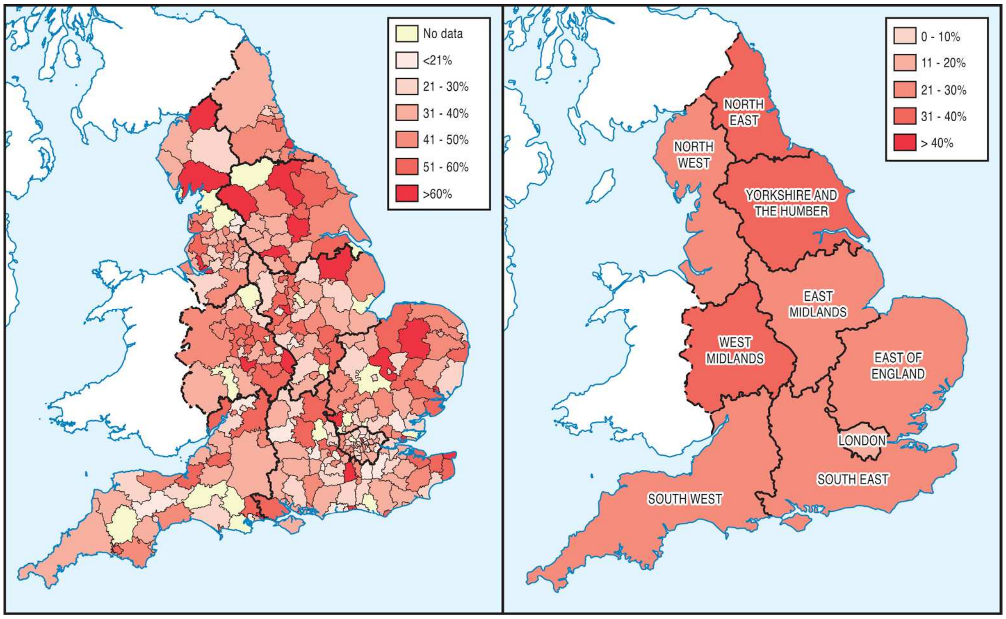

Figure 1: Prevalence of chronic pain by local authority and English region

122x74mm (600 x 600 DPI)

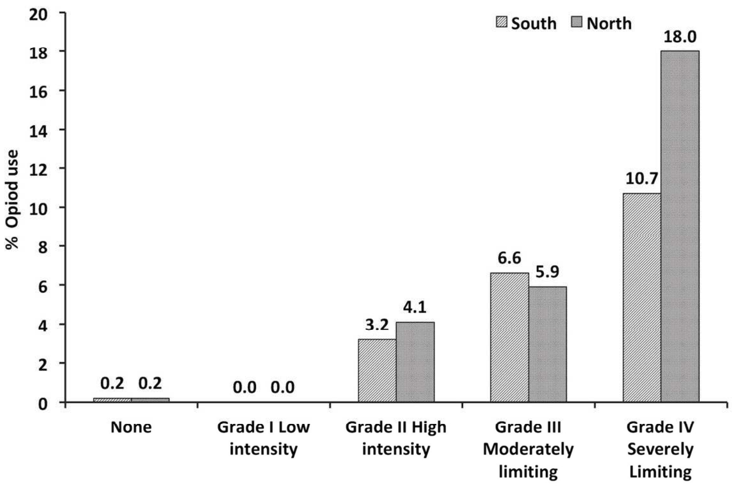

Figure 2: Opioid use among participants from the North and South of England according to chronic pain grades

208x117mm (300 x 300 DPI)

STROBE Statement—checklist of items that should be included in reports of observational studies

	Item No.	Recommendation	Page No.	Relevant text from manuscript
**Title and abstract**	1	(*a*) Indicate the study's design with a commonly used term in the title or the abstract	1	
		(*b*) Provide in the abstract an informative and balanced summary of what was done and what was found	2-3	
**Introduction**				
Background/rationale	2	Explain the scientific background and rationale for the investigation being reported	5	
Objectives	3	State specific objectives, including any prespecified hypotheses	7	
**Methods**				
Study design	4	Present key elements of study design early in the paper	7	
Setting	5	Describe the setting, locations, and relevant dates, including periods of recruitment, exposure, follow-up, and data collection	7, 8	
Participants	6	(*a*) *Cohort study*—Give the eligibility criteria, and the sources and methods of selection of participants. Describe methods of follow-up<br>*Case-control study*—Give the eligibility criteria, and the sources and methods of case ascertainment and control selection. Give the rationale for the choice of cases and controls<br>*Cross-sectional study*—Give the eligibility criteria, and the sources and methods of selection of participants	7-9	
		(*b*) *Cohort study*—For matched studies, give matching criteria and number of exposed and unexposed<br>*Case-control study*—For matched studies, give matching criteria and the number of controls per case		
Variables	7	Clearly define all outcomes, exposures, predictors, potential confounders, and effect modifiers. Give diagnostic criteria, if applicable	8 and Tables 2 and 3	
Data sources/ measurement	8*	For each variable of interest, give sources of data and details of methods of assessment (measurement). Describe comparability of assessment methods if there is more than one group	8	
Bias	9	Describe any efforts to address potential sources of bias	9 (no missing data)	
Study size	10	Explain how the study size was arrived at	NA – it was	

For peer review only - http://bmjopen.bmj.com/site/about/guidelines.xhtml

For peer review only

	taken from HSE

Continued on next page

For peer review only - http://bmjopen.bmj.com/site/about/guidelines.xhtml

Quantitative variables	11	Explain how quantitative variables were handled in the analyses. If applicable, describe which groupings were chosen and why	9
Statistical methods	12	(*a*) Describe all statistical methods, including those used to control for confounding	9
		(*b*) Describe any methods used to examine subgroups and interactions	9
		(*c*) Explain how missing data were addressed	NA (there was none)
		(*d*) *Cohort study*—If applicable, explain how loss to follow-up was addressed<br>*Case-control study*—If applicable, explain how matching of cases and controls was addressed<br>*Cross-sectional study*—If applicable, describe analytical methods taking account of sampling strategy	NA
		(*e*) Describe any sensitivity analyses	NA
**Results**			
Participants	13*	(a) Report numbers of individuals at each stage of study—eg numbers potentially eligible, examined for eligibility, confirmed eligible, included in the study, completing follow-up, and analysed	9 (gives numbers in HSE)
		(b) Give reasons for non-participation at each stage	NA – secondary data
		(c) Consider use of a flow diagram	NA – secondary data
Descriptive data	14*	(a) Give characteristics of study participants (eg demographic, clinical, social) and information on exposures and potential confounders	12, Table 1
		(b) Indicate number of participants with missing data for each variable of interest	NA)
		(c) *Cohort study*—Summarise follow-up time (eg, average and total amount)	NA
Outcome data	15*	*Cohort study*—Report numbers of outcome events or summary measures over time	NA
		Case-control study—Report numbers in each exposure category, or summary measures of exposure	NA
		Cross-sectional study—Report numbers of outcome events or summary measures	Table 2, and 3
Main results	16	(*a*) Give unadjusted estimates and, if applicable, confounder-adjusted estimates and their precision (eg, 95% confidence interval). Make clear which confounders were adjusted for and why they were	Table 2 and 3

For peer review only - http://bmjopen.bmj.com/site/about/guidelines.xhtml

	included	
	(*b*) Report category boundaries when continuous variables were categorized	NA
	(*c*) If relevant, consider translating estimates of relative risk into absolute risk for a meaningful time period	NA

Continued on next page

For peer review only

For peer review only - http://bmjopen.bmj.com/site/about/guidelines.xhtml

Other analyses	17	Report other analyses done—eg analyses of subgroups and interactions, and sensitivity analyses	NA
**Discussion**			
Key results	18	Summarise key results with reference to study objectives	16
Limitations	19	Discuss limitations of the study, taking into account sources of potential bias or imprecision. Discuss both direction and magnitude of any potential bias	18-19
Interpretation	20	Give a cautious overall interpretation of results considering objectives, limitations, multiplicity of analyses, results from similar studies, and other relevant evidence	16-17
Generalisability	21	Discuss the generalisability (external validity) of the study results	17-18
**Other information**			
Funding	22	Give the source of funding and the role of the funders for the present study and, if applicable, for the original study on which the present article is based	20

*Give information separately for cases and controls in case-control studies and, if applicable, for exposed and unexposed groups in cohort and cross-sectional studies.

Note: An Explanation and Elaboration article discusses each checklist item and gives methodological background and published examples of transparent reporting. The STROBE checklist is best used in conjunction with this article (freely available on the Web sites of PLoS Medicine at http://www.plosmedicine.org/, Annals of Internal Medicine at http://www.annals.org/, and Epidemiology at http://www.epidem.com/). Information on the STROBE Initiative is available at www.strobe-statement.org.

For peer review only

BMJ Open

The Pain Divide: A cross-sectional analysis of chronic pain prevalence, pain intensity, and opioid utilisation in England

Journal:	*BMJ Open*
Manuscript ID	bmjopen-2018-023391.R1
Article Type:	Research
Date Submitted by the Author:	01-May-2018
Complete List of Authors:	Todd, Adam; Newcastle University<br>Akhter, Nasima; Durham University, Wolfson Research Institute for Health and Wellbeing<br>Cairns, Joanne; Newcastle University, Institute of Health and Society<br>Kasim, Adetayo; Durham University, Wolfson Research Institute for Health and Wellbeing<br>Walton, Nick; Newcastle University, Institute of Health and Society<br>Ellison, Amanda; Durham University<br>Chazot, Paul; Durham University<br>Eldabe, Sam; South Tees NHS Trust<br>Bambra, Clare; Newcastle University
**Primary Subject Heading**:	Public health
Secondary Subject Heading:	Addiction
Keywords:	PAIN MANAGEMENT, PUBLIC HEALTH, Health policy < HEALTH SERVICES ADMINISTRATION & MANAGEMENT

SCHOLARONE™
Manuscripts

The Pain Divide: A cross-sectional analysis of chronic pain prevalence, pain intensity, and opioid utilisation in England

Adam Todd*[1,2,3], reader in pharmaceutical public health adam.todd@newcastle.ac.uk
Nasima Akhter[2,4], assistant professor (research) nasima.akhter@durham.ac.uk
Joanne-Marie Cairns[1,2,5], lecturer in public health, j_m_cairns@hotmail.co.uk
Adetayo Kasim,[2,4], associate professor (research) a.s.kasim@durham.ac.uk
Nick Walton[1,2,3], research associate, n.walton2@newcastle.ac.uk
Amanda Ellison[4,6], reader in psychology amanda.ellison@durham.ac.uk
Paul Chazot[4,7], associate professor in pharmacology paul.chazot@durham.ac.uk
Sam Eldabe[1,8], consultant anesthesia and pain medicine Sam.Eldabe@stees.nhs.uk
Clare Bambra[1,2], professor of public health, clare.bambra@newcastle.ac.uk

[1]Institute of Health and Society, Faculty of Medical Sciences, Newcastle University, Baddiley-Clark building, Newcastle upon Tyne, NE2 4AX, UK.
[2]Fuse – the UKCRC Centre for Translational Research in Public Health, UK.
[3]School of Pharmacy, Faculty of Medical Sciences, Newcastle University, King's Road, Newcastle upon Tyne, NE1 7RU, UK.
[4]Wolfson Research Institute for Health and Wellbeing, Durham University, Queen's Campus, Stockton-on-Tees, TS17 6BH, UK.
[5] School of Public Health Midwifery and Social Work, Canterbury Christchurch University, Canterbury, CT1 1QU, UK
[6]Department of Psychology, Durham University, South Road, Durham, DH1 3LE, UK.
[7]Department of Biosciences, Durham University, South Road, Durham, DH1 3LE, UK.
[8]Department of Pain and Anaesthesia, The James Cook University Hospital, Marton Rd, Middlesbrough, TS4 3BW, UK.

*Dr. Adam Todd is the corresponding author; adam.todd@newcastle.ac.uk +44 (0) 191 208 2355

For peer review only

For peer review only - http://bmjopen.bmj.com/site/about/guidelines.xhtml

Abstract

Objectives

Our central research question was, in England, are geographical inequalities in opioid use driven by health need (pain)? To answer this question, our study examined: (1) if there are regional inequalities in rates of chronic pain prevalence, pain intensity and opioid utilisation in England; (2) if opioid use and chronic pain are associated after adjusting for individual and area level confounders.

Design

Cross-sectional study design using data from the Health Survey for England 2011

Setting

England

Primary and secondary outcome measures

Chronic pain prevalence, pain intensity, and opioid utilization

Participants

Participant data relating to chronic pain prevalence, pain intensity, and opioid usage data were obtained at local authority level from the Health Survey for England 2011; in total 5711 respondents were included in our analysis.

Methods

Regional and local authority data were mapped, and a generalised linear model was then used to explore the relationships between the data. The model was adjusted to account for area and individual level variables.

For peer review only - http://bmjopen.bmj.com/site/about/guidelines.xhtml

For peer review only

Results

There were geographical variations in chronic pain prevalence, pain intensity, and opioid utilisation across the English regions – with evidence of a 'pain divide' between the North and the South, whereby people in the North of England more likely to have 'severely limiting' or 'moderately limiting' chronic pain. The intensity of chronic pain was significantly, and positively associated with the use of opioid analgesics.

Conclusions

There are geographical differences in chronic pain prevalence, pain intensity, and opioid utilisation across England – with evidence of a 'pain-divide'. Given the public health concerns associated with the long-term use of opioid analgesics – and their questionable activity in the management of chronic pain – more guidance is need to support prescribers in the management of long chronic pain so the initiation of opioids can be avoided.

Keywords: opioids, chronic pain, public health, addiction, appropriate prescribing

Word count of main manuscript: 3432

For peer review only

For peer review only - http://bmjopen.bmj.com/site/about/guidelines.xhtml

Strengths and limitations of this study

- This study is unique in that we explored the association of opioid utilisation and chronic pain
- We adjusted for individual (e.g. age and sex) and area-level confounders (e.g. social deprivation) in our model.
- We did not distinguish between weak and strong opioid in our analysis, nor did we consider dose of opioid

For peer review only

For peer review only - http://bmjopen.bmj.com/site/about/guidelines.xhtml

Introduction

Chronic pain is a worldwide problem, and the burden it places on our society is increasing: in the US, the annual cost of chronic pain – through direct and indirect effects – is estimated to exceed $500 billion, while in the UK estimates suggest it costs around £12 billion per year to the economy.[1, 2] To manage the symptoms associated with chronic pain, some treatment strategies rely on the use of opioid analgesics, although there are very few studies to support their long-term effectiveness.[3-5] In addition, prolonged use of opioids can also have adverse consequences; this can include sleep disturbances, endocrine disorders, reduced immune function and increased pain through opioid-induced hyperalgesia.[6-10]

Despite these well-acknowledged shortcomings, the prescribing of opioid analgesics continues to increase at a significant rate.[11-12] Indeed, figures from the UK show that, in 2014, there were around 23 million prescriptions written for opioid analgesics, at a cost of around £322 million.[13] Given this increased use, (and the well-established problems associated with efficacy, tolerance and adverse effects) the inappropriate prescribing – and misuse – of opioid analgesics is becoming a significant public health concern.[14] This problem is also mirrored in other countries, such as the US, where the death rate from opioid misuse has, in the last 15 years, quadrupled – giving rise to the so-called 'opioid epidemic'.[15]

In England, there is significant geographical variation in opioid prescribing – with more people in the North of England prescribed opioids – at a greater cost – compared to the rest of England. For example, the North of England (population of 15 million) accounts for approximately 33 per cent of the total costs of analgesics, compared to London (population of 8.2 million), that accounts for only around 8 per cent.[12] It is not clear, however, if this variation is related to 'inappropriate prescribing' or the varying health need of the population (*i.e.* more people in the North of England have pain, hence the prescribing of opioids is

For peer review only - http://bmjopen.bmj.com/site/about/guidelines.xhtml

higher). It is well documented, though, that mortality and morbidity rates are higher in the North of England, particularly in the North-East region compared to the rest of England: an observation known as the North South health divide.[16]

Northern England (commonly defined as the North East, North West and Yorkshire and Humber regions) has persistently had higher all-cause mortality rates than the South of England, with people in the North consistently found to be less healthy than those in the South - across all social classes and amongst men and women.[17] Since 1965, this has amounted to 1.5 million excess premature deaths.[18] Further, the gap in average life expectancy gap between the North and the South of England is 2 years.[16] Although England is not alone in experiencing such spatial health inequalities, the divide in England is one of the largest in Europe – greater, for example, than those between the former East and West of Germany.[19] Social science suggests that the reasons for the contemporary health divide are both compositional and contextual.[16] Compositional factors include demographic factors (e.g. age, sex, marital status) and socio-economic status (e.g. employment, income, education, occupation), as well as health behaviours (e.g. smoking, alcohol, physical activity). In the case of pain, other compositional factors will include co-morbidities such as depression or anxiety. Contextual factors include the physical (e.g. air pollution or contaminated land),[20] social (e.g. place based stigma or social networks or access to services such as GPs)[21] and economic (e.g. area-level deprivation, local job availability) environments.[22]

Given the North South health divide and public health concerns associated with the inappropriate and long-term use of opioid analgesics, it is vitally important then to explore whether the prescribing of opioid analgesics across England reflects inequalities in the health needs of the population (pain) or if there is an issue of ‘inappropriate’ medication prescribing or utilisation. Our central research question therefore was, in England, are geographical

For peer review only - http://bmjopen.bmj.com/site/about/guidelines.xhtml

inequalities in opioid use driven by health need (pain)? To answer this question, our study examined: (1) if there are regional inequalities in rates of chronic pain prevalence, pain intensity and opioid utilisation in England; (2) if opioid use and chronic pain are associated after adjusting for individual and area level confounders.

Methods

Data and Variables

Local authority level Health Survey for England (HSE) data were obtained from the National Centre for Social Research, which contains anonymised individual-level data and a geographic identifier (Local Authority District which are large administrative areas used by local government in England and have the responsibility for health and social care, education, transport and so forth). The HSE is an annual survey designed to be representative using a stratified random sample. Each year there is a focus on a particular population group, condition or disease. In 2011, one particular focus of the HSE was detailing chronic pain: as part of the wider survey, participants were asked:

- Whether they were currently troubled by pain or discomfort?
- Whether they had this pain or discomfort for more than 3 months?

If the respondent answered yes to both questions, they were categorised as experiencing chronic pain. Once it was established that participants had chronic pain, they were then asked a further three questions:

- How would you rate your pain right now, on a scale from 0 to 10, where 0 is no pain and 10 is pain as bad as it could be?
- In the last three months, how would you rate your worst pain, on a scale from 0 to 10, where 0 is no pain and 10 is pain as bad as it could be?

For peer review only - http://bmjopen.bmj.com/site/about/guidelines.xhtml

- On average, in the last three months, how would you rate your pain on a scale from 0 to 10, where 0 is no pain and 10 is pain as bad as it could be?

The answers to these questions were then used to compute a variable on pain intensity on a scale of 0 to 4, indicating 'grade 0 - no intensity (i.e. no chronic pain)', 'grade 1 – low intensity', 'grade 2 – high intensity', grade 3 – moderately limiting', 'grade 4 – severely limiting'. This grading was based upon the 3-item Graded Chronic Pain (GCP)-Pain Catastrophizing Scale (PCS).[23]

Opioid usage data was also contained in the 2011 HSE; this used the British National Formulary (BNF) classification code for opioid analgesic medications. Socio-demographic variables included were age, sex, marital status, highest educational qualifications, occupational classifications, household income quintile. Health related data included self-assessed general health status (very good; good; fair; bad; very bad), presence of mental health disorder (yes / no), anxiety levels (not anxious or depressed; moderately anxious or depressed; extremely anxious or depressed), and ranking of happiness on a 0 to 10 scale. Area-level deprivation data included the Index of Multiple Deprivation (IMD) 2010 obtained from the HSE. The IMD produces a ranking of areas in England based on relative local scores for: income, employment, health, education, crime, access to services and living environment. IMD was included because there is a strong relationship between area level deprivation and mortality and morbidity – with the most deprived neighbourhoods in England experiencing life expectancy nine and six years less for men and women respectively than those that are the least deprived.[24]

The English regions were classified as the North (North East, North West, Yorkshire & the Humber) and the South (London, East of England, West Midlands, East Midlands, South East

For peer review only

For peer review only - http://bmjopen.bmj.com/site/about/guidelines.xhtml

and South West). This study used individual level HSE data and therefore HSE survey weights applicable for individual level data were used.

Data Analysis

Chronic pain prevalence, pain intensity, and opioid usage were mapped using Adobe Illustrator with local and regional boundaries downloaded from the Office for National Statistics. In the HSE, opioid use was described as a binary variable (a yes or no response), and was used as an outcome variable to examine the association between opioid use and factors associated with it. The HSE 2011 individual level data had 10617 cases, and pain data were only collected among respondents aged 16 years and over (n = 8610). Cases where there were missing values for the confounding variables (regions, age, sex, marital status, highest educational qualification, occupational level, household income quintiles, general health status, mental health disorders, anxiety levels, and happiness scale) were then excluded from our analysis. Missing values in the HSE can occur for several reasons, including refusal or inability to answer a particular question or refusal to co-operate in an entire section of the survey. After this, the dataset with no missing values (n = 5711) was used in our analysis. Variables that showed significantly bivariate association were included in the initial model. Apart from the presence of chronic pain and pain intensity, the initial model included age, sex, marital status, highest educational qualification, occupational level, household income quintiles, general health status, mental health disorders, anxiety levels, and happiness scale. A generalised linear model with binomial distribution and logit link was used to examine the associations between opioid use and chronic pain, adjusted for individual and area level covariates. Survey weight was applied to the model. The most parsimonious model was obtained by using likelihood ratio test statistics to ensure there was no significant loss of information. To support the spatial analysis of a 'pain-divide' between the North and the South of England, the pain intensity data were analysed using a generalised logit model to

For peer review only - http://bmjopen.bmj.com/site/about/guidelines.xhtml

simultaneously analyse the four logit models resulting from the five levels of the pain intensity data (no pain, low intensity, high intensity, moderately limiting and severely limiting). Although the pain intensity is ordinal, the proportional odds model is both intuitively and statistically not appropriate because of the assumption of the common odds between the levels of pain intensity data. Survey weight was used in all analyses to ensure generalization of findings.

This study was undertaken and reported according to the strengthening the reporting of observational studies in epidemiology (STROBE) recommendations.[25] Data analysis was done using SAS 9.4.

Patient and Public Involvement

As this study involved secondary data analysis from the HSE, patients or the public were not involved in the design, or delivery of this research.

Ethical approval

Ethical approval of this work was not required, as the study used non-patient identifiable secondary data; patients were not actively involved in this research.

Results

Regional inequalities in the prevalence of chronic pain, pain intensity and opioid use in England

The prevalence of chronic pain was 39.6% in the North of England, compared to the 37.5% in the South of England, as shown in Table 1, and visually in Figure 1. In terms of the nine English regions, the prevalence of chronic pain was highest in the North East, and lowest in

For peer review only - http://bmjopen.bmj.com/site/about/guidelines.xhtml

London (43.1% vs. 29.0%). In terms of pain intensity, 10.2% of people living in the South had ‘moderately limiting’ or ‘severely limiting’ chronic pain, while, in the North, 13.9% of people had ‘moderately limiting’ or ‘severely limiting’ chronic pain. People in the North were also more likely to experience ‘moderately limiting’ or ‘severely limiting’ pain than those in the South: the odds of severely limiting pain were 32% higher in the North than in the South; similarly, the odds of ‘moderately limiting’ pain were 37% higher in the North than the South, as shown in Table 2. In addition to differing pain levels in the North and South English regions, there were also observed differences in anxiety and self-reported general health: anxiety levels in the North were 27.3%, compared to 25.7% in the South, while for self-reported general health, 7.6% and 5.5% of people living in the North and South respectively were reported to have ‘bad’ or ‘very bad’ health status. Although chronic pain prevalence was similar in the North and South of England (39.6% and 37.5%, respectively), opioid use was somewhat higher in the North (3.0%), compared to the South (1.9%). Furthermore, the use of opioids (weighted results) were higher in the North of England for people with ‘severely limiting’ chronic pain (17%), compared to people in the South (10%), as illustrated by Figure 2.

For peer review only

For peer review only - http://bmjopen.bmj.com/site/about/guidelines.xhtml

Table 1. Characteristics of the study population

Variable		South	North	Overall
		% (n)	% (n)	% (n)
**Region**		68.2 (3896)	31.8 (1815)	100.0 (5711)
**Age group**	Median (25th, 75th percentile)	45 (32, 60)	45 (32, 60)	45 (32, 60)
**Sex**	Female	55.9 (2178)	55.3 (1003)	55.7 (3181)
	Male	44.1 (1718)	44.7 (812)	44.3 (2530)
**Marital status**	Single	25.5 (995)	27.7 (503)	26.2 (1498)
	Married/ civil partner	55.8 (2173)	50.7 (921)	54.2(3094)
	Divorced/ widowed/ separated	18.7 (728)	21.5 (391)	19.6 (1119)
**Anxiety grades**	Extreme	2.2 (85)	2.9 (53)	2.4 (138)
	Moderate	23.5 (917)	24.4 (443)	23.8 (1360)
	Not anxious	74.3 (2894)	72.7 (1319)	73.8 (4213)
**Income quintiles**	Highest	25.1 (977)	16.4 (297)	22.3 (1274)
	Second highest	22.7 (884)	20.2 (367)	21.9 (1251)
	Middle	19.9 (775)	20.6 (374)	20.1 (1149)
	Second lowest	18.2 (708)	25.1 (455)	20.4 (1163)
	Lowest	14.2 (552)	17.7 (322)	15.3 (874)
**Occupation**	Managerial and professional	40.2 (1566)	34.2 (620)	38.3 (2186)
	Intermediate	25.2 (982)	21.5 (390)	24.0 (1372)
	Routine and manual	31.0 (1209)	40.9 (743)	34.2 (1952)
	Other	3.6 (139)	3.4 (62)	3.5 (201)
**Educational qualifications**	No qualifications	17.3 (674)	21.8 (395)	8.7 (1069)
	Foreign/ other	1.4 (54)	1.5 (27)	1.4 (81)
	NVQ1 or equivalent	4.0 (155)	4.7 (86)	4.2 (241)
	NVQ2 or equivalent	22.2 (864)	22.0 (399)	22.1 (1263)
	NVQ3/ A level equivalent	15.6 (608)	15.4 (279)	15.5 (887)
	Higher education	11.9 (462)	12.2 (222)	12.0 (684)
	NVQ4/ Degree or equivalent	27.7 (1079)	22.4 (407)	26.0 (1488)
**General health**	Very bad	1.3 (52)	2.3 (42)	1.6 (94)
	Bad	4.2 (165)	5.3 (96)	4.6 (261)
	Fair	15.6 (607)	18.2 (330)	16.4 (937)
	Good	44.1 (1719)	42.8 (776)	43.7 (2495)
	Very good	34.7 (1353)	31.5 (571)	33.7 (1924)
**Mental health disorder**	No condition	96.0 (3741)	95.6 (1735)	95.9 (5476)
	Has condition	4.0 (155)	4.4 (80)	4.1 (235)
**Happiness scale**	Median (25th, 75th percentile)	8 (7,9)	8 (7,9)	8 (7, 9)

Weighted results with confidence intervals and numbers

Opioid use	No	98.3 (97.9, 98.7; n =3897)	97.5 (96.8, 98.3; n = 1577	98.1 (97.7; 98.5, n = 5474)
	Yes	1.7 (1.3, 2.1; n = 66)	2.5 (1.7, 3.3; n= 40)	1.9 (1.5, 2.3; n = 106)

For peer review only

For peer review only - http://bmjopen.bmj.com/site/about/guidelines.xhtml

**Chronic Pain intensity**	None	65.0 (63.5, 66.5; n= 2574)	63.3 (60.9, 65.6; n = 1024)	64.5 (63.2, 65.7; n = 3598)
	Low intensity	1.7 (1.3, 2.1; n = 66)	1.4 (0.8, 1.9; n= 22)	1.6 (1.3, 1.9; n = 88)
	High intensity	24.2 (22.8, 25.5; n = 958)	23.0 (21.0, 25.1; n = 373)	23.8 (22.7, 24.9; n = 1331)
	Moderately limiting	3.4 (2.8, 3.9; n =134)	4.3 (3.3, 5.3; n= 69)	3.6 (3.1, 4.1; n = 203)
	Severely limiting	5.8 (5.1, 6.6; n =231)	8.0 (6.7, 9.4; n= 130)	6.5 (5.8, 7.1; n= 361)

For peer review only

For peer review only - http://bmjopen.bmj.com/site/about/guidelines.xhtml

Table 2. Estimated odds ratios from generalised logit analysis of different pain intensities between North and South of England adjusting for age, gender and level of qualifications.

Variables	Categories	'Severely limiting' Vs 'No Pain'	P-value	'Moderately limiting' Vs 'No Pain'	P-value	'High Intensity' Vs 'No Pain'	P-value	'Low Intensity' Vs 'No Pain'	P value
Intercept		0.011 (0.007,0.018)	<0.001	0.010 (0.006,0.018)	<0.001	0.097 (0.076,0.124)	<0.001	0.004 (0.002,0.010)	<0.001
Region	North	1.323 (1.063,1.645)	0.012	1.374 (1.035,1.823)	0.028	0.977 (0.852,1.120)	0.735	0.954 (0.604,1.507)	0.840
	South	Ref		Ref		Ref		Ref	
Age		1.042 (1.035,1.050)	<0.001	1.033 (1.024,1.042)	<0.001	1.030 (1.025,1.034)	<0.001	1.036 (1.022,1.054)	<0.001
Gender	Female	1.137 (1.020,1.267)	0.021	1.221 (1.059,1.408)	0.006	1.183 (1.108,1.262)	<0.001	0.913 (0.738,1.129)	0.399
	Male	Ref		Ref		Ref		Ref	
Qualifications	None	2.574 (2.069,3.203)	<0.001	1.408 (1.021,1.943)	0.037	1.133 (0.964,1.332)	0.131	1.117 (0.660,1.890)	0.681
	Foreign/ other qualification	1.188 (0.635,2.222)	0.591	0.873 (0.351,2.171)	0.770	0.894 (0.574,1.392)	0.619	1.373 (0.384,4.911)	0.626
	NVQ1 CSE other grade equivalent	1.345 (0.872,2.077)	0.180	1.082 (0.587,1.995)	0.801	1.199 (0.917,1.569)	0.185	0.562 (0.164,1.922)	0.358
	NVQ2 GCE O level equivalent	1.057 (0.822,1.358)	0.667	1.073 (0.776,1.483)	0.669	1.007 (0.868,1.169)	0.927	0.748 (0.422,1.326)	0.320
	NVQ3 GCE A level equivalent	0.853 (0.621,1.171)	0.324	0.744 (0.487,1.138)	0.173	0.913 (0.768,1.087)	0.307	0.866 (0.459,1.636)	0.658
	Higher education below degree	0.718 (0.513,1.005)	0.054	0.937 (0.627,1.401)	0.752	0.999 (0.836,1.194)	0.991	1.162 (0.654,2.065)	0.609
	NVQ4/NVQ5/ Degree	Ref		Ref		Ref		Ref	

For peer review only

For peer review only - http://bmjopen.bmj.com/site/about/guidelines.xhtml

Association of opioid utilisation and chronic pain after adjusting for individual and area level confounders

Opioid usage was significantly associated with chronic pain intensity (adjusted for age, household income, occupation level, general health and anxiety): in people with higher pain intensities, there were higher odds of opioid use, as illustrated by Table 3. The use of opioids were also positively associated with household income levels: households belonging to the 3rd to 5th (highest) income quintiles had significantly higher odds of using opioids than those at the lowest quintile. In addition, general health status was significantly positively associated with opioid usage: people who reported 'very bad' or 'bad' health status had 14% higher odds, and 6% higher odds of using opioids respectively, compared to those who reported 'very good' health status. Finally, participants who reported extreme anxiety or depression had significantly higher odds of using opioid analgesics, compared to participants who were not anxious.

For peer review only - http://bmjopen.bmj.com/site/about/guidelines.xhtml

Table 3. Generalised Linear Model examining associations between opioid use and chronic pain

Variables	Categories	Odds Ratio (Confidence Intervals)	P-values
Intercept		0.970 (0.956, 0.985)	<0.001
Age		1.000 (1.000, 1.000)	0.112
Pain grade	Severely limiting	1.078 (1.060, 1.097)	<0.001
	Moderately limiting	1.036 (1.016, 1.056)	<0.001
	High intensity	1.022 (1.013, 1.031)	<0.001
	Low intensity	0.995 (0.968, 1.023)	0.746
	No chronic pain	Ref	
Income quintile	Highest quintile	1.017 (1.004, 1.030)	0.008
	4th	1.018 (1.006, 1.030)	0.004
	3rd	1.018 (1.006, 1.030)	0.003
	2nd	1.016 (1.004, 1.028)	0.007
	Lowest quintile	Ref	
Highest qualification	No qualification	1.012 (1.000, 1.024)	0.059
	Foreign/ other	1.005 (0.972, 1.039)	0.761
	NVQ1 or equivalent	1.002 (0.984, 1.021)	0.809
	NVQ2 or equivalent	1.004 (0.994, 1.015)	0.400
	NVQ3/ A level equivalent	1.011 (1.000, 1.022)	0.050
	Higher education	1.005 (0.992, 1.017)	0.461
	NVQ4/ Degree or equivalent	Ref	
General Health	Very bad	1.137 (1.102, .174)	<0.001
	Bad	1.057 (1.035, 1.080)	<0.001
	Fair	1.022 (1.010, 1.034)	<0.001
	Good	1.000 (0.992, 1.008)	0.995
	Very good	Ref	
Anxiety	Extreme	1.015 (0.991, 1.039)	0.227
	Moderate	1.008 (1.000, 1.017)	0.052
	Not anxious	Ref	

For peer review only

For peer review only - http://bmjopen.bmj.com/site/about/guidelines.xhtml

Discussion

This paper is the first to examine geographical inequalities in chronic pain prevalence, pain intensity, and opioid utilisation in England. It is also the first to examine the association between chronic pain intensity and opioid utilisation. We have identified two key findings that may be of importance to healthcare practitioners and policy makers: (1) there are geographical variations in chronic pain prevalence, pain intensity, and opioid utilisation across the English regions – with evidence of a 'pain-divide' with people in the North of England more likely to have higher intensity of pain; (2) opioid utilisation was significantly, and positively associated with pain intensity. The higher prevalence and intensity of pain in the Northern regions, as well as more people to lower education groups may only partly explain the higher rates of opioid usage found there. However, the number of people who used opioids in the survey was too small to support an interaction model between pain intensity and regions or a separate subgroup analysis for each region. These findings suggest the reason why people in the North East of England are prescribed more opioid analgesics than other parts of England is owing to the higher health need (pain). This is in keeping with wider studies of regional inequalities in health [16] and is a potentially important and significant finding given the recent public health concerns associated with opioid analgesics.

While this is the first study to examine the relationship between chronic pain intensity and opioid usage in England, there have been other studies that have explored the geographical variation in opioid prescribing. For example, a recent study by Mordecai and colleagues showed that, at a clinical commissioning group (CCG) level, over a four-year period, there was an increasing trend of opioid prescribing – with more opioid analgesics prescribed in the North of England, compared to the South.[26] Our work builds on these findings, and shows that the increased trend of opioid prescribing is associated with an increase in health need (pain), rather than an 'inappropriate' prescribing trend of opioid analgesics. In addition to

For peer review only - http://bmjopen.bmj.com/site/about/guidelines.xhtml

this, there have been a number of studies that have explored prescribing variation in other parts of the world, such the US,[27, 28] Canada,[29] and Australia;[30] these studies have also showed there is a large geographical variation in prescribing practices of opioid analgesics, and call for guidance to promote good prescribing practices. Our results are timely, and show that, in England, the prescribing of opioid analgesics is largely driven by health need (pain): thus, to develop future strategies going forward, and avoid a potential 'opioid epidemic', as observed in the US, it is important that consideration is given to other ways of managing chronic pain, without the use of opioid analgesics. While opioids may have a role in the short-term management of pain, their long-term use is questionable.[6-10] Currently, national guidelines recommend strong opioids as an option for pain relief for patients with chronic pain, providing they are reviewed annually, and only continued if they are providing on-going pain relief.[31] While this is helpful in some instances, it is often difficult to ascertain, in a clinical setting, if opioid analgesics continue to provide on-going pain relief; patients using opioids are also often reluctant to reduce or stop their opioid medication.[32,33] Studies also show that opioid discontinuation is associated with reducing pain scores; opioid induced hyperalgesia also reduces upon opioid cessation, which can further reduce levels of pain.[34] Given our findings, more needs to be done – at a national level – to support prescribers to manage people who have chronic pain, without the need to initiate opioid analgesics. Another potential that could be potentially used alongside this approach would be to consider how opioids are monitored and stopped in the community. We note the recent attention given to the term 'deprescribing'– a term used to describe the process of reducing or stopping inappropriate medication, with a view to minimising polypharmacy and improving patient outcomes.[35] It would be prudent to suggest that future prescribing strategies for opioids should also include an element of 'deprescribing' to ensure

For peer review only

For peer review only - http://bmjopen.bmj.com/site/about/guidelines.xhtml

that if opioids are to be initiated, patients do not continue to use or be prescribed opioids for chronic pain indefinitely without benefit.

Our findings relating to geographical inequalities in chronic pain are in keeping with research into a number of other health outcomes, such as obesity, diabetes, cancer and cardiovascular disease, where higher rates are reported in the North – and in particular the North East – compared to the other English regions.[16] Our work suggests that the North South health divide could increase in the future unless prescribing practices change because current guidance for using opioids to manage pain means that the North will have a higher burden of side effects in the future. Further, with an ageing population (particularly in the North) and an associated increase in chronic conditions, then we anticipate a further increase in pain and therefore opioid use. Again, given the regional inequalities in the burden of disease, this could exacerbate further the North South divide. This is timely, as the recent Due North report,[18] an independent inquiry, commissioned by Public Health England, to identify actions that can reduce the gap in health between the North and South of England suggests that an urgent holistic approach is needed to ensure that future investment is effective at reducing inequalities. Our study shows that examination of the need for continued opioid prescribing should be considered in any strategies going forward to tackle the poorer health outcomes commonly reported in the North East of England, compared to the rest of the country.

In terms of study limitations, we acknowledge that there are several: firstly, in our analysis we used chronic pain prevalence and pain intensity as the marker for health need. Opioids are also used in the management of other conditions, such as acute post-operative pain, cancer pain, or in the management of opioid substance dependence; clearly, this will have an influence regarding opioid prescribing practices. Also, the analysis does not discriminate between specific opioids, potency of opioid (*e.g.* strong opioids versus weak opioids) or

For peer review only - http://bmjopen.bmj.com/site/about/guidelines.xhtml

opioid dosages. It is also important to consider that geographical scale is important when exploring variation amongst a given area: it is possible that, even at Local Authority level, the opioid prevalence estimates are concealing further geographical patterning since they still contain relatively large populations. A finer scale analysis may, therefore, highlight particular opioid 'hotspots' where opioid prescribing and utilisation is concentrated. Another study limitation is that the HSE data was from 2011, although we note this is the most recent and meaningful data on chronic pain prevalence and pain intensity. Finally, the usual limitations of using cross-sectional data apply to this study meaning that we cannot claim causation only association, nor can we say that this association applies at other geographical scales, only at a regional level. While we believe our results are robust, and have important policy implications, they should be interpreted cautiously in view of our acknowledged limitations.

Conclusion

There are geographical differences in chronic pain prevalence, pain intensity, and opioid utilisation across England – with evidence of a 'pain-divide' with people in the North of England more likely to have 'severely limiting' or 'moderately limiting' chronic pain. In our model, the intensity of chronic pain was significantly, and positively associated with the use of opioid analgesics. Given the public health concerns associated with the long-term use of opioid analgesics – and their questionable activity in the management of chronic pain – more guidance is need to support prescribers in the management of long chronic pain so the initiation of opioid can be avoided. Future opioid prescribing strategies should also consider incorporating deprescribing approaches to ensure when opioids are initiated, their use is regularly monitored, reviewed and, discontinued in the community.

For peer review only - http://bmjopen.bmj.com/site/about/guidelines.xhtml

Figure/Table legends

Figure 1: Prevalence of chronic pain by local authority and English region

Figure 2: Opioid use among participants from the North and South of England according to chronic pain grades

Table 1: Characteristics of the study population

Table 2: Estimated odds ratios from generalised logit analysis of different pain intensities between North and South of England adjusting for age, gender and level of qualifications.

Table 3. Generalised linear model of associations between opioid use and chronic pain

Contributions

AT, CB and SE designed the study, and supervised all stages of the research. AT led the drafting of the manuscript with input from all authors. AK and NA led the statistical analyses; NW cleaned the data, conducted preliminary analyses and commented on the drafts. CB, AT, and JC led on data interpretation. AE and PC informed the initial study design and commented on the analysis, and interpretation. AT is the corresponding author and acts as guarantor of the article.

Competing interests

The authors declare that they have no competing interests.

Funding

This research received no specific grant from any funding agency in the public, commercial or not-for-profit sectors.

For peer review only

For peer review only - http://bmjopen.bmj.com/site/about/guidelines.xhtml

Ethical approval and consent to participate

Ethical approval of this work was not required, as the study used non-patient identifiable secondary data; patients were not actively involved in this research.

Acknowledgements

We would like to acknowledge the National Centre for Social Research for supplying us with the sub-national HSE data required for the analysis. The Wolfson Research Institute for Health & Wellbeing supported us financially with a small grant to purchase the data. Chris Orton, Durham University, in Cartography produced the map in this paper, and we thank him for his assistance. Author CB is a member of Fuse. Funding for Fuse comes from the British Heart Foundation, Cancer Research UK, Economic and Social Research Council, Medical Research Council, the National Institute for Health Research, under the auspices of the UK Clinical Research Collaboration, and is gratefully acknowledged (RF150334). The views expressed in this paper do not necessarily represent those of the funders or UKCRC. The funders had no role in study design, data collection and analysis, decision to publish, or preparation of the manuscript.

Data sharing agreement

Unfortunately, we are unable to share our data, as it does not belong to us. We have an agreement with HSCIC (source of our secondary data) that we will delete the data once we are finished using it.

For peer review only - http://bmjopen.bmj.com/site/about/guidelines.xhtml

References:

1. Relieving Pain in America: A Blueprint for Transforming Prevention, Care, Education, and Research. Institute of Medicine (US) Committee on Advancing Pain Research, Care, and Education. Washington (DC): National Academies Press (US); 2011

2. Maniadakis N, Gray A. The economic burden of back pain in the UK. *Pain.* 2000; 84(1): 95-103.

3. Azevedo L, Costa-Pereira A, Mendonça L, Dias C, Castro-Lopes J. A population-based study on chronic pain and the use of opioids in Portugal. *Pain.* 2013; 154(12): 2844-52.

4. Jensen M, Thomsen A, Højsted J. 10-year follow-up of chronic non-malignant pain patients: opioid use, health related quality of life and health care utilization. *Eur J Pain.* 2006; 10(5): 423-33.

5. Stannard C. Opioids for chronic pain: promise and pitfalls. *Curr Opin Support Palliat Care.* 2011; 5: 150-7.

6. Webster L, Choi Y, Desai H, Webster L, Grant B. Sleep-disordered breathing and chronic opioid therapy. *Pain Med.* 2008;9(4):425-32.

7. Wang D, Teichtahl H. Opioids, sleep architecture and sleep-disordered breathing. Sleep Med Rev. 2007;11(1):35-46.

8. Asaad TA, Ghanem MH, Abdel Samee, AM, El–Habiby, MM. Sleep Profile in Patients With Chronic Opioid Abuse: A Polysomnographic Evaluation in an Egyptian Sample. *Addictive Disorders & Their Treatment.* 2011;10(1):21-8.

For peer review only - http://bmjopen.bmj.com/site/about/guidelines.xhtml

9. Martell B, O'Connor P, Kerns R, Becker W, Morales K, Kosten T, *et al.* Systematic review: opioid treatment for chronic back pain: prevalence, efficacy, and association with addiction. *Ann Intern Med.* 2007; 146(2): 116-27.

10. Berna C, Kulich R, Rathmell J. Tapering Long-term Opioid Therapy in Chronic Noncancer Pain: Evidence and Recommendations for Everyday Practice. *Mayo Clin Proc.* 2015; 90(6): 828-42.

11. Zin C, Chen L-C, Knaggs R. Changes in trends and pattern of strong opioid prescribing in primary care. *Eur J Pain.* 2014; 18(9): 1343–51.

12. Murphy E, Spain V. General Practice Prescribing Trends: 2014 Annual Review. London: Cogora, 2015.

13. Team PaM. Prescriptions dispensed in the community: England, 2004 to 2014: Health and Social Care Information Centre; 2015. Available from: http://www.hscic.gov.uk/catalogue/PUB17644/pres-disp-com-eng-2004-14-rep.pdf (last accessed 04.03.2018)

14. Schmidt TD, Haddox JD, Nielsen AE, Wakeland W, Fitzgerald J. Key Data Gaps Regarding the Public Health Issues Associated with Opioid Analgesics. *J Behav Health Serv Res.* 2015; 42(4): 540-53.

15. Calcaterra S, Glanz J, Binswanger IA. National trends in pharmaceutical opioid related overdose deaths compared to other substance related deaths: 1999-2009. *Drug Alcohol Depend.* 2013; 131(3): 263-70.

16. Bambra, C. Health Divides: Where You Live Can Kill You. Policy Press. 2016. ISBN: 978-1447330356.

For peer review only - http://bmjopen.bmj.com/site/about/guidelines.xhtml

17. Dorling D 2010 *Persistent North-South Divides* in Coe, NM Jones A eds The Economic Geography of the UK Sage Publications London.

18. Whitehead M (chair), Bambra C, Barr B, Bowles J, Caulfield R, Doran T, Harrison D, Lynch A, Pleasant S, and Weldon, J. (2014) *Due North: The Independent Inquiry into Health Equity in the North* CLES: Manchester. (http://www.cles.org.uk/publications/due-north-report-of-the-inquiry-on-health-equity-for-the-north/) (last accessed 04.03.2018)

19. Bambra C, Barr B, Milne E. 2014 North and South: addressing the English health divide. *J Public Health (Oxf)*. 2014;36(2):183-6.

20. Bambra C, Cairns JM, Kasim A, Smith J, Robertson S, Copeland A, Johnson K. (2015) This divided land: An examination of regional inequalities in exposure to brownfield land and the association with morbidity and mortality in England. *Health Place.* 2015;34:257-69.

21. Todd A, Copeland A, Kasim A, Husband A, Bambra C. Access all areas? An area-level analysis of the relationship between community pharmacy and primary care distribution, urbanity and social deprivation in England, *BMJ Open*. 2015;5:e007328.

22. Macintyre S, Ellaway A, Cummins S. Place effects on health: how can we conceptualise, operationalise and measure them? *Soc Sci Med.* 2002;55(1):125-39.

23. Sullivan MJL. The Pain Catastrophizing Scale (PCS); user manual. Available at: http://sullivan-painresearch.mcgill.ca/pdf/pcs/PCSManual_English.pdf (last accessed 04.03.2018)

24. Inequality in healthy life expectancy at birth by national deciles of area deprivation: England. Office for National Statistics. Available at: https://www.ons.gov.uk/peoplepopulationandcommunity/healthandsocialcare/healthandlifeex

For peer review only - http://bmjopen.bmj.com/site/about/guidelines.xhtml

pectancies/datasets/inequalityinhealthylifeexpectancyatbirthbynationaldecilesofareadeprivationengland (last accessed 04.03.2018)

25. STROBE statements. Available at: https://strobe-statement.org/index.php?id=available-checklists (last accessed 04.03.2018)

26. Mordecai L, Reynolds C, Donaldson LJ, Williams A. Patterns of regional variation of opioid prescribing in primary care in England: a retrospective observational study. *Br J Gen Pract* 2018; DOI: https://doi.org/10.3399/bjgp18X695057.

27. McDonald DC, Carlson K, Izrael D. Geographic variation in opioid prescribing in the U.S. *J Pain.* 2012; 13(10): 988-96.

28. Kuehn BM. CDC: Major disparities in opioid prescribing among states: some states crack down on excess prescribing. *JAMA.* 2014; 312(7): 684-6.

29. Gomes T, Juurlink D, Moineddin R, Gozdyra P, Dhalla I, Paterson M, Mamdani M. Geographical variation in opioid prescribing and opioid-related mortality in Ontario. *Healthc Q.* 2011;14(1):22-4.

30. Degenhardt L, Gisev N, Cama E, Nielsen S, Larance B, Bruno R. The extent and correlates of community-based pharmaceutical opioid utilisation in Australia. *Pharmacoepidemiol Drug Saf.* 2016; 25(5):521-38.

31. SIGN 136. Management of Chronic Pain. Available at: http://www.sign.ac.uk/pdf/SIGN136.pdf (last accessed 04.03.2018)

32. Kennedy LC, Binswanger IA, Mueller SR, Levy C, Matlock DD, Calcaterra SL, Koester S, Frank JW. "Those Conversations in My Experience Don't Go Well": A Qualitative Study

For peer review only

For peer review only - http://bmjopen.bmj.com/site/about/guidelines.xhtml

of Primary Care Provider Experiences Tapering Long-term Opioid Medications. *Pain Med.* 2017; doi: 10.1093/pm/pnx276.

33. Frank JW, Levy C, Matlock DD, Calcaterra SL, Mueller SR, Koester S, Binswanger IA. Patients' Perspectives on Tapering of Chronic Opioid Therapy: A Qualitative Study. *Pain Med.* 2016;17(10):1838-1847.

34. Sullivan MD, Turner JA, DiLodovico C, D'Appollonio A, Stephens K, Chan YF. Prescription Opioid Taper Support for Outpatients With Chronic Pain: A Randomized Controlled Trial. *J Pain.* 2017;18(3):308-318.

35. Scott IA, Hilmer SN, Reeve E, Potter K, Le Couteur D, Rigby D, Gnjidic D, Del Mar CB, Roughead EE, Page A, Jansen J, Martin JH. Reducing inappropriate polypharmacy: the process of deprescribing. *JAMA Intern Med.* 2015; 175(5): 827-34.

For peer review only

For peer review only - http://bmjopen.bmj.com/site/about/guidelines.xhtml

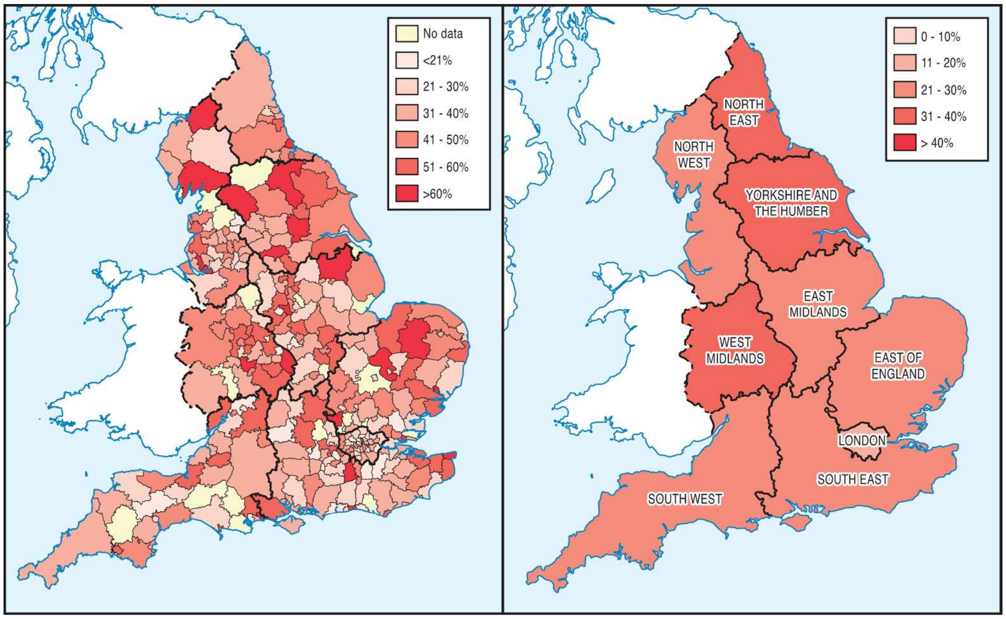

Figure 1: Prevalence of chronic pain by local authority and English region

122x74mm (600 x 600 DPI)

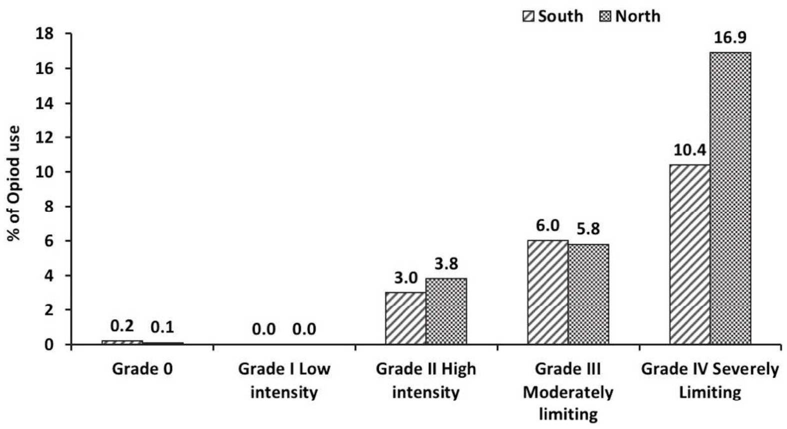

Figure 2: Opioid use among participants from the North and South of England according to chronic pain grades

337x189mm (300 x 300 DPI)

review only

STROBE Statement—checklist of items that should be included in reports of observational studies

	Item No.	Recommendation	Page No.	Relevant text from manuscript
**Title and abstract**	1	(*a*) Indicate the study's design with a commonly used term in the title or the abstract	1	
		(*b*) Provide in the abstract an informative and balanced summary of what was done and what was found	2-3	
**Introduction**				
Background/rationale	2	Explain the scientific background and rationale for the investigation being reported	5	
Objectives	3	State specific objectives, including any prespecified hypotheses	7	
**Methods**				
Study design	4	Present key elements of study design early in the paper	7	
Setting	5	Describe the setting, locations, and relevant dates, including periods of recruitment, exposure, follow-up, and data collection	7, 8	
Participants	6	(*a*) *Cohort study*—Give the eligibility criteria, and the sources and methods of selection of participants. Describe methods of follow-up<br>*Case-control study*—Give the eligibility criteria, and the sources and methods of case ascertainment and control selection. Give the rationale for the choice of cases and controls<br>*Cross-sectional study*—Give the eligibility criteria, and the sources and methods of selection of participants	7-9	
		(*b*) *Cohort study*—For matched studies, give matching criteria and number of exposed and unexposed<br>*Case-control study*—For matched studies, give matching criteria and the number of controls per case		
Variables	7	Clearly define all outcomes, exposures, predictors, potential confounders, and effect modifiers. Give diagnostic criteria, if applicable	8 and Tables 2 and 3	
Data sources/ measurement	8*	For each variable of interest, give sources of data and details of methods of assessment (measurement). Describe comparability of assessment methods if there is more than one group	8	
Bias	9	Describe any efforts to address potential sources of bias	9 (no missing data)	
Study size	10	Explain how the study size was arrived at	NA – it was	

For peer review only - http://bmjopen.bmj.com/site/about/guidelines.xhtml

	taken from HSE

Continued on next page

For peer review only

For peer review only - http://bmjopen.bmj.com/site/about/guidelines.xhtml

Quantitative variables	11	Explain how quantitative variables were handled in the analyses. If applicable, describe which groupings were chosen and why	9
Statistical methods	12	(*a*) Describe all statistical methods, including those used to control for confounding	9
		(*b*) Describe any methods used to examine subgroups and interactions	9
		(*c*) Explain how missing data were addressed	NA (there was none)
		(*d*) *Cohort study*—If applicable, explain how loss to follow-up was addressed<br>*Case-control study*—If applicable, explain how matching of cases and controls was addressed<br>*Cross-sectional study*—If applicable, describe analytical methods taking account of sampling strategy	NA
		(*e*) Describe any sensitivity analyses	NA
**Results**			
Participants	13*	(a) Report numbers of individuals at each stage of study—eg numbers potentially eligible, examined for eligibility, confirmed eligible, included in the study, completing follow-up, and analysed	9 (gives numbers in HSE)
		(b) Give reasons for non-participation at each stage	NA – secondary data
		(c) Consider use of a flow diagram	NA – secondary data
Descriptive data	14*	(a) Give characteristics of study participants (eg demographic, clinical, social) and information on exposures and potential confounders	12, Table 1
		(b) Indicate number of participants with missing data for each variable of interest	NA)
		(c) *Cohort study*—Summarise follow-up time (eg, average and total amount)	NA
Outcome data	15*	*Cohort study*—Report numbers of outcome events or summary measures over time	NA
		Case-control study—Report numbers in each exposure category, or summary measures of exposure	NA
		Cross-sectional study—Report numbers of outcome events or summary measures	Table 2, and 3
Main results	16	(*a*) Give unadjusted estimates and, if applicable, confounder-adjusted estimates and their precision (eg, 95% confidence interval). Make clear which confounders were adjusted for and why they were	Table 2 and 3

For peer review only - http://bmjopen.bmj.com/site/about/guidelines.xhtml

For peer review only

	included	
	(*b*) Report category boundaries when continuous variables were categorized	NA
	(*c*) If relevant, consider translating estimates of relative risk into absolute risk for a meaningful time period	NA

Continued on next page

For peer review only

For peer review only - http://bmjopen.bmj.com/site/about/guidelines.xhtml

Other analyses	17	Report other analyses done—eg analyses of subgroups and interactions, and sensitivity analyses	NA
**Discussion**			
Key results	18	Summarise key results with reference to study objectives	16
Limitations	19	Discuss limitations of the study, taking into account sources of potential bias or imprecision. Discuss both direction and magnitude of any potential bias	18-19
Interpretation	20	Give a cautious overall interpretation of results considering objectives, limitations, multiplicity of analyses, results from similar studies, and other relevant evidence	16-17
Generalisability	21	Discuss the generalisability (external validity) of the study results	17-18
**Other information**			
Funding	22	Give the source of funding and the role of the funders for the present study and, if applicable, for the original study on which the present article is based	20

*Give information separately for cases and controls in case-control studies and, if applicable, for exposed and unexposed groups in cohort and cross-sectional studies.

Note: An Explanation and Elaboration article discusses each checklist item and gives methodological background and published examples of transparent reporting. The STROBE checklist is best used in conjunction with this article (freely available on the Web sites of PLoS Medicine at http://www.plosmedicine.org/, Annals of Internal Medicine at http://www.annals.org/, and Epidemiology at http://www.epidem.com/). Information on the STROBE Initiative is available at www.strobe-statement.org.

For peer review only

For peer review only - http://bmjopen.bmj.com/site/about/guidelines.xhtml
